# No evidence of carbapenemase-producing Enterobacteriaceae in stool samples of 1,544 asylum seekers arriving in Rhineland-Palatinate, Germany, April 2016 to March, 2017

**DOI:** 10.2807/1560-7917.ES.2019.24.8.1800030

**Published:** 2019-02-21

**Authors:** Lutz Ehlkes, Yvonne Pfeifer, Guido Werner, Ralf Ignatius, Manfred Vogt, Tim Eckmanns, Philipp Zanger, Jan Walter

**Affiliations:** 1 Federal State Agency for Consumer & Health Protection Rhineland-Palatinate, Koblenz, Germany; 2 Postgraduate Training for Applied Epidemiology (PAE), Robert Koch Institute, Berlin, Germany; 3 European Programme for Intervention Epidemiology Training (EPIET), European Centre for Disease Prevention and Control (ECDC), Stockholm, Sweden; 4 Robert Koch Institute, Nosocomial Pathogens and Antibiotic Resistance, Wernigerode, Germany; 5 MVZ Labor 28, Berlin, Germany; 6 Institute of Microbiology, Charité - Universitätsmedizin Berlin, Campus Benjamin Franklin, Berlin, Germany; 7 Robert Koch Institute, Nosocomial Infections and Surveillance of Antibiotic Resistance, Berlin, Germany; 8 Heidelberg Institute of Global Health, Unit of Epidemiology and Biostatistics, University Hospitals, Heidelberg, Germany; 9 Department of Infectious Diseases, Medical Microbiology and Hygiene, University Hospitals, Heidelberg, Germany; 10 These two authors have contributed equally to this manuscript and share last authorship

**Keywords:** carbapenem-resistant Enterobacteriaceae, Escherichia coli, ESBL, beta-lactamase CTX-M-27, beta-lactamase CTX-M-15, E. coli ST131, Syria, Afghanistan, Iran, Pakistan, Eritrea, Somalia, human migration, mass screening, refugees, drug resistance, microbial, communicable diseases, emerging, epidemiology, infectious disease transmission, prevalence, cross-sectional studies, plasmid mediated fluoroquinolone resistance

## Abstract

Introduction: Since 2015, increased migration from Asia and Africa to Europe has raised public health concerns about potential importation of extended-spectrum β-lactamase-producing Enterobacteriaceae (ESBL-PE), specifically those producing carbapenemases (C-PE), into European hospitals.

Aims: To inform infection control practices about ESBL-PE prevalence in asylum seekers and to investigate whether C-PE prevalence exceeds that in the German population.

Methods: Cross-sectional study from April 2016–March 2017. Routinely collected stool samples from asylum seekers were tested for antibiotic resistant Enterobacteriaceae. Country/region of origin and demographic characteristics were explored as risk factors for faecal colonisation.

Results: Of 1,544 individuals, 294 tested positive for ESBL-PE colonisation (19.0%; 95% confidence intervals (CI): 17.0–21.0). Asylum seekers originating from Afghanistan/Pakistan/Iran had a prevalence of 29.3% (95% CI: 25.6–33.2), from Syria 20.4% (95% CI: 16.1–25.2) and from Eritrea/Somalia 11.9% (95% CI: 8.7–15.7). CTX-M-15 (79%) and CTX-M-27 (10%) were the most common ESBL determinants. Highest ESBL-PE prevalences were observed in boys under 10 years and women aged 20–39 years (interaction: p = 0.03). No individuals tested positive for C-PE. Faecal C-PE colonisation prevalence in asylum seekers was not statistically significantly different from prevalence reported in German communities.

Conclusion: In absence of other risk factors, being a newly arrived asylum seeker from a region with increased faecal ESBL-PE colonisation prevalence is not an indicator for C-PE colonisation and thus not a reason for pre-emptive screening and isolation upon hospital admission.

## Introduction

Antibiotic resistance of pathogens and resulting limitations of therapeutic options increase morbidity, mortality and costs [1]. Since the beginning of this century, the number of infections caused by extended-spectrum β-lactamase-producing Enterobacteriaceae (ESBL-PE) has grown rapidly. Of these, carbapenemase-producing Enterobacteriaceae (C-PE) are of particular interest, as carbapenems are considered compounds of last resort against life-threatening infections. Major steps in the spread of antibiotic resistance in Enterobacteriaceae are horizontal exchange of mobile resistance genes into different clones and their dissemination over long distances, often facilitated by travel or migration of the colonised host [2]. International travel to south/south-east Asia and Africa was found to be a risk factor for colonisation [3,4] and subsequent infection with ESBL-PE [5]. Introduction of these bacteria into unaffected hospitals is much dreaded since nosocomial outbreaks of C-PE have been reported world-wide [6].

In 2015, Europe was challenged by the arrival of a large number of asylum seekers, sparked primarily by the Syrian civil war, but also by other conflicts and humanitarian crises in southern Asia, western Asia, and Africa. Reports of high ESBL-PE and C-PE colonisation prevalences in hospitalised populations and among asylum seekers from these countries/regions [7-12], together with data on increased ESBL-PE and C-PE colonisation in returning travellers [3,4] led to discussions whether such migration may increase the risk of nosocomial transmission of multidrug-resistant bacteria in European countries with low C-PE prevalence [13].

In April 2016, the European Centre for Disease Prevention and Control (ECDC) recommended that individuals with recent exposure in high prevalence countries may also be considered for pre-emptive screening and isolation upon admission in European hospitals, even if they had no history of hospitalisation or antibiotic therapy before their arrival in Europe [14]. However, published studies regarding ESBL-PE/C-PE colonisation of migrants seeking asylum in the European Union are limited to research in hospitalised patients [9,10,15,16], do not stratify by country/region of origin [9,10,15,16], and lack statistical power [10,11,15]. Thus, while these studies provide rough estimates of the ESBL-PE/C-PE colonisation prevalence in populations that have *a priori* an increased likelihood of pre-morbidities, they do not represent newly arrived asylum seekers in general and thus cannot inform whether country/region of origin alone is a sufficient predictor for increased risk of ESBL-PE/C-PE colonisation. Therefore, research on colonisation status in sufficiently large populations recruited outside health care institutions is needed to determine whether newly arrived asylum seekers in general require pre-emptive screening and isolation upon hospital admission [14].

This study aimed to determine the colonisation prevalence of ESBL-PE/C-PE in asylum seekers newly arrived to the federal state of Rhineland-Palatinate, Germany, and to compare whether the prevalence of colonisation with C-PE exceeds that reported in the German community [17].

## Methods

We conducted a cross-sectional study from April 2016–March 2017, on the prevalence of ESBL-PE and C-PE in stool samples from asylum seekers arriving in the federal state Rhineland-Palatinate, Germany.

### Study population

According to federal state law, a medical examination including the analysis of one stool sample is mandatory for each individual within 1 week of seeking asylum [18]. Upon arrival in one of the 29 refugee reception centres in Rhineland-Palatinate, each asylum seeker is provided with a sample tube, pictogram and pre-paid packaging to be sent to the federal state public health laboratories for further processing. There, native stool samples are routinely tested for *Salmonella* spp., *Shigella* spp. and helminth eggs [19]. Stool samples from asylum seekers were split into a routine and a study aliquot, given there was sufficient material. We recorded country of origin (self-reported), year of birth and sex from routinely collected data in pseudo-anonymised form using an eight digit number code. Samples from 42 asylum seekers providing incomplete personal information (n=22) or insufficient material (n=20) were excluded. Using data from the first 6 months, we decided to restrict sampling to subjects from the most frequently occurring regions of origin: Syria (Western Asia), Afghanistan, Pakistan and Iran (all South Asia), as well as Eritrea and Somalia (both East Africa).

### Sample size

We powered our study to provide evidence against the null hypothesis: ‘there is no difference in the prevalence of C-PE in asylum seekers arriving in Rhineland-Palatinate compared to that of the German community’. We used population prevalence estimates based on a study from Valenza et al. [17], who reported one C-PE in 3,344 individuals (0.03%; 95% CI: 0.00–0.17) in a community-based sample in Germany. Based on this, we considered a prevalence of 0.20% or higher in asylum seekers to be of public health relevance. Using 80% power, an α-error of 5%, and a one-sided comparison against a fixed population value, we calculated to enrol 1,512 individuals using the ‘sampsi’ command in Stata 14 (StataCorp LP, College Station, Texas, United States of America). A maximum period of 12 months was predetermined in case the desired sample size would not be met.

### Microbiological analyses

Stool samples were sent to a diagnostic laboratory (Labor 28 GmbH, Berlin, Germany) for isolation of Enterobacteriaceae and ESBL phenotyping. All samples were spread on chromogenic screening agar (Brilliance ESBL agar, Oxoid, Wesel, Germany). Single colonies of different morphotypes were further cultured and bacterial species were identified using the Vitek 2 system (bioMérieux, Nürtingen, Germany). Susceptibilities to amoxicillin, amoxicillin/clavulanic acid, mezlocillin, piperacillin, piperacillin/tazobactam, tigecycline, cefuroxime, cefotaxime, cefepime, ceftazidime, cefpodoxime, ceftriaxone, imipenem, meropenem, ertapenem, gentamicin, tobramycin, ciprofloxacin, moxifloxacin, levofloxacin, trimethoprim and trimethoprim/sulfamethoxazole were determined and the results interpreted according to the European Committee on Antimicrobial Susceptibility Testing (EUCAST) breakpoints [20]. All isolates with ESBL phenotype (resistance to cefotaxime and/or ceftazidime and/or ESBL phenotype according to the automated expert system implemented in Vitek 2 system) were sent to the Robert Koch Institute, Wernigerode, Germany, for molecular analyses.

Presence of various β-lactamase genes (*bla*_VIM-like_, *bla*_NDM-like_, *bla*_OXA-48-like_, *bla*_NDM-like_, *bla*_CTX-M-1–2-9group_, *bla*_TEM-like_, *bla*_SHV-like_, *bla*_CMY-like_, *bla*_OXA-1-group_,) was tested by PCR and sequencing using previously described primers [21-23]. Additionally, a PCR screening for plasmid-mediated genes contributing to resistance to fluoroquinolones (*aac(6’)Ib-cr*, *qnrA/B/S-like*) was performed as described [24,25]. For PCR screening of *qnrC* and *qnrD* genes, the following primers were used: *qnrC* fwd 5’- atttccaaggggcaaactg-3’and *qnrC* rev 5’-aactgctccaaaagctgctc-3’(amplification product 400bp), *qnrD* fwd 5’-ttgtgatttttcaggggttg-3’ and *qnrD* rev 5’- cctgctctccatccaacttc-3’ (amplification product 521bp). Possible presence of plasmid-mediated colistin resistance genes *mcr-1* and *mcr-2* was tested by PCR as described [26,27]. For *Escherichia coli *isolates, the PCR-based identification of the four main phylogenetic groups was performed as described [28]. Furthermore, the proportion of *E. coli* multilocus sequence type (ST) ST131 was determined by PCR-based assays identifying the clonal lineages *E. coli* O25b:H4-ST131 and O16:H5-ST131 [29,30].

### Data analysis and statistics

All records were stored in an EpiData database and imported into Stata 14. Countries were grouped by region according to United Nations (UN) standard M49 [31]. Age was categorised in steps of 10 years, with individuals aged 40 years and over being combined in the ‘40+’ group. Based on the binomial distribution, exact confidence intervals (CI) were calculated for prevalence estimates, using 95% CI as default and one-sided 97.5% CI for the upper limit of zero frequencies.

To determine potential risk factors for ESBL-PE/C-PE colonisation, we analysed the influence of age, sex and geographic origin on colonisation using cross tabulation as well as uni- and multivariable logistic regression modelling. We allowed for interaction between the explanatory variables and kept the interaction term, if a likelihood ratio test indicated improved model fit.

### Ethical considerations

The study protocol was reviewed and approved by the ethics commission, board of physicians, Federal State of Rhineland-Palatinate, Germany (ref. number 837.487.15).

## Results

In total, 1,544 individuals were screened, of whom 955 (62%) were male and 589 (38%) were female; the median age was 23 years for both men (inter quartile range: 17–32 years) and women (inter quartile range 16–34 years). Asylum seekers from Syria were the most prominent group (n = 324), followed by Afghanistan (n = 282), Eritrea (n = 192), Iran (n = 170), Somalia (n = 161), and Pakistan (n = 115). Bacterial growth on ESBL screening agar was detectable for stool samples from 297 individuals. Three non-Enterobacteriaceae isolates (*Pseudomonas aeruginosa, Pseudomonas putida,* and *Acinetobacter baumannii*) with third generation cephalosporin resistance, but neither carbapenem resistance nor carbapenem non-susceptibility were found in stool samples of two individuals and were subsequently excluded from the study.

**Figure 1:**
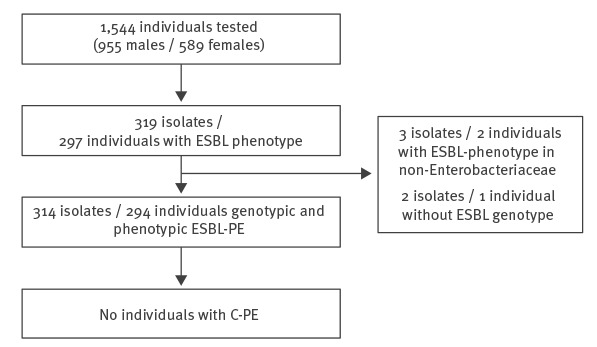
Description of study sample from newly arrived asylum seekers, Germany, April 2016–March 2017

In total, 316 isolates of Enterobacteriaceae with ESBL phenotype (310 *E. coli*, three *Klebsiella pneumoniae*, two *Morganella morganii*, and one *Citrobacter freundii*) were detected in stool samples from 295 individuals. Twenty-one of these individuals supplied stool samples, which contained two different isolates (20 contained different *E. coli* morphotypes, one contained *E. coli* and *M. morganii*). In two of the 316 isolates (*M. morganii, C. freundii*), no ESBL genes could be detected. A subsequently performed disk test for ESBL/AmpC production (D68C ESBL/AmpC ID MAST-group) confirmed both isolates as AmpC producers, probably due to species-specific chromosomal encoded enzymes. Thus, both isolates (one individual) were excluded, leaving 314 isolates from 294 individuals for the analysis of phenotypic and genotypic resistance (Figure 1).

ESBL: extended-spectrum β-lactamase; ESBL-PE: extended-spectrum β-lactamase-producing Enterobacteriaceae; C-PE: carbapenemase producing Enterobacteriaceae.

ESBL phenotype defined as specific growth on chromogenic screening agar.

### C-PE and ESBL-PE colonisation

**Figure 2:**
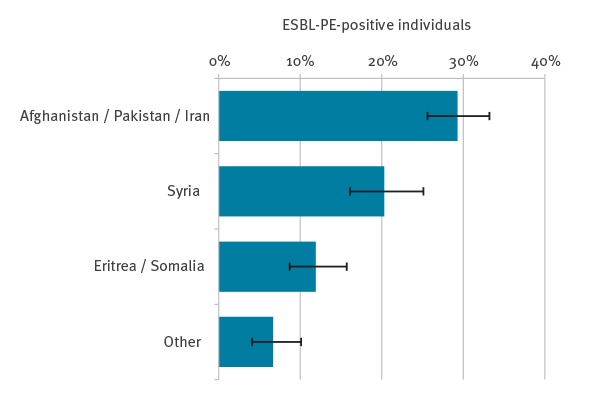
Prevalence of ESBL-PE colonisation among asylum seekers, by country/region of origin, Germany, April 2016–March 2017 (n = 1,544)

Our final sample included 1,544 individuals, of whom 294 tested positive for ESBL-PE (19.0%, 95% CI: 17.0–21.0), with a total of 314 isolates containing ESBL genes (310 *E. coli*, three *K. pneumoniae*, one *M. morganii*) (Figure 1). Stratifying the results showed that prevalence of ESBL-PE colonisation varied by county/region of origin (chi-squared p < 0.001). Highest prevalences were observed in individuals from Afghanistan/Pakistan/Iran 29.3% (n = 166/567; 95% CI: 25.6–33.2), followed by Syria 20.4% (66/324; 95% CI: 16.1–25.2), and Eritrea/Somalia 11.9% (42/353; 95% CI: 8.7–15.7). Samples combined in the “Other” category included 300 individuals (ESBL-PE positive: n = 20 (6.7%); 95% CI: 4.1–10.1), originating from the following countries: Albania (n = 0/48), Bosnia-Herzegovina (n = 2/25), The Republic of North Macedonia (n = 3/23), Armenia (n = 1/18), Azerbaijan (n = 2/16), Serbia (n = 0/11), Kosovo* (n = 0/10), as well as 149 individuals (12 positive) from countries with less than 10 samples each (Figure 2).

ESBL-PE: extended-spectrum β-lactamase-producing Enterobacteriaceae.

Bars represent 95% confidence intervals. Sample size by category was Afghanistan/Pakistan/Iran (n = 166/567), Syria (n = 66/324), Eritrea/Somalia (n = 42/353), other (n = 20/300). The ‘Other’ category contains results from subjects from Albania (n = 0/48), Armenia (n = 1/18), Azerbaijan (n = 2/16), Bosnia-Herzegovina (n = 2/25), Kosovo* (n = 0/10), The Republic of North Macedonia (n = 3/23), Serbia (n = 0/11) and from 149 individuals (12 positive) from countries with less than 10 samples each. P value < 0.001 from χ2 test comparing proportion of ESBL-PE-positive individuals over strata of origin.

*This designation is without prejudice to positions on status, and is in line with UNSCR 1244/1999 and the ICJ Opinion on the Kosovo declaration of independence.

**Figure 3:**
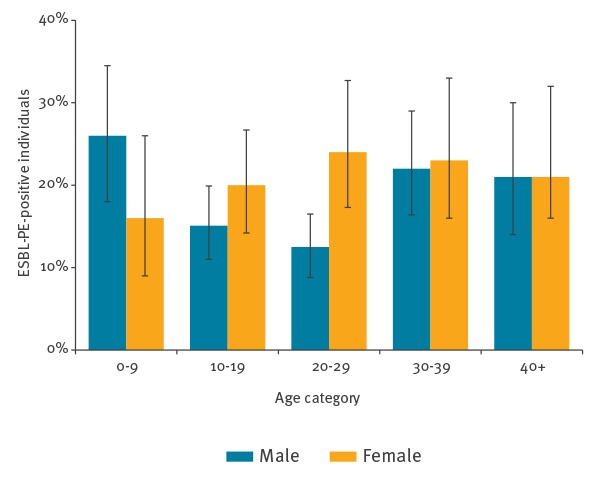
Prevalence of ESBL-PE colonisation among asylum seekers, by age category and sex, Germany, April 2016–March 2017 (n = 1,544)

As shown in Figure 3, prevalence of ESBL colonisation varied by age group and sex, with a range over categories of age and sex from 13% to 27%. Highest prevalences were observed in boys under 10 years (25.6%; 95% CI: 18.0–34.5), and in women aged 20–29 years (24.4%; 95% CI: 17.3–32.7) and 30–39 years (23.2%; 95% CI: 15.8–32.1). A likelihood ratio test comparing multivariable regression models of the association of sex and age with ESBL-prevalence showed better model fit when allowing for interaction between age and sex (p value = 0.03) (Figure 3).

ESBL-PE: extended-spectrum β-lactamase-producing Enterobacteriaceae.

Bars represent 95% confidence intervals.

Likelihood ratio test for interaction between age and sex gives a p value of 0.03.

No C-PE were detected in any of the samples, resulting in a prevalence estimate of 0.0% (upper 97.5% confidence limit 0.2%) for the overall population of asylum seekers. There was no evidence to reject the null hypothesis of C-PE in asylum seekers being equal to that previously reported in the German population (fixed population prevalence 0.20%, p value = 0.75). Based on sample size and according precision in estimating the proportion of C-PE in respective strata, the 0% C-PE prevalence estimates had upper one-sided 97.5% confidence limits of 1.1% for Syria, 0.6% for Afghanistan/Pakistan/Iran, and 1.0% for Eritrea/Somalia, respectively.

### Antibiotic resistance

The 314 isolates with ESBL genes (310 *E. coli*, three *K. pneumoniae*, one *M. morganii*) were resistant to penicillines and 3^rd^ generation cephalosporins. Furthermore, they showed additional resistance to ciprofloxacin, gentamicin, and trimethoprim-sulfamethoxazole in 33% (n = 103), 16% (n = 51), and 63% (n = 199), respectively. Intermediate susceptibility to tigecycline was detected in two *K. pneumoniae* isolates.

### Genetic determinants of antibiotic resistance

Table 1 summarises the results of the molecular characterisation of ESBL-PE, and Table 2 shows additional β-lactamase and plasmid-mediated quinolone resistance (PMQR) genes, stratified by geographic region. The two most prevalent ESBL genes were *bla*_CTX-M-15_ (n=249; 79%) and *bla*_CTX-M-27_ (n=30; 10%). Four isolates (1.3%) with ESBL gene *bla*_CTX-M-15_ ESBL carried additional *ampC* genes (*bla*_CMY-4_, *bla*_CMY-42_, *bla*_CMY-58_, and *bla*_CMY-59)_. β-lactamase genes *bla*_TEM-like_ and *bla*_OXA-1_ were found in 125 (39.8%) and 43 (13.7%) of the ESBL producing isolates, respectively. The three *K. pneumoniae* isolates carried different *bla*_SHV_ genes (*bla*_SHV-1,_ *bla*_SHV-11,_ *bla*_SHV-75_) in combination with ESBL gene *bla*_CTX-M-15_. A substantial amount of the ESBL-PE isolates were positive for the *qnrS1 gene* (81/314, 25.8%) contributing to fluoroquinolone resistance; other variants (*qnrB1,* 3/314, 1.0%; *qnrB19,* 3/314, 1.0%; *qnrA1,* 1/314, 0.3%) and methytransferase gene *aac(6’)Ib-cr* (40/314, 12.7%) were found in small numbers. Plasmid-mediated colistin resistance genes *mcr-1* or *mcr-2* were not found in any of the ESBL-PE.

**Table 1:** Extended-spectrum β-lactamases in Enterobacteriaceae from 1,544 newly arrived asylum seekers, by country/region of origin, Germany, April 2016–March 2017 (n = 314)

ESBL	Total N = 314		Country/region of origin							
			Pakistan/Afghanistan/Iran^a^ n = 183		Syria n = 68		Eritrea/Somalia^a^ n = 42		Other^b^ n = 21	
	n	%	n	%	n	%	n	%	n	%
CTX-M-15	249	79	158	86	51	75	30	71	10	48
CTX-M-27	30	10	15	8	5	7	4	10	6	29
CTX-M-1	7	2	1	1	4	6	2	5	0	0
CTX-M-3	8	3	2	1	4	6	1	2	1	5
CTX-M-55	6	2	4	2	0	0	2	5	0	0
SHV-12	4	1	1	1	0	0	1	2	2	10
CTX-M-14	4	1	0	0	2	3	1	2	1	5
CTX-M-24	2	1	1	1	1	1	0	0	0	0
CTX-M-9	1	0	0	0	1	1	0	0	0	0
CTX-M-17	1	0	0	0	0	0	0	0	1	5

ESBL: Extended-spectrum β-lactamase.

^a^ Grouped according to United Nations Regions M49 standard [31].

^b^ Includes isolates from countries with less than five positive stool samples.

Data are number (column %) of detected ESBL genes (PCR and sequencing) in Enterobacteriaceae from asylum seekers (total N), by country of origin, unless indicated otherwise. P value for chi-squared test of the null hypothesis: ‘Presence of any of these extended spectrum β-lactamase genes in ESBL isolates is equally distributed over countries/regions of origin’ is < 0.001.

**Table 2:** β-lactamase- and plasmid-mediated quinolone resistance genes in ESBL-producing Enterobacteriaceae from 1,544 newly arrived asylum seekers, by country/region of origin, Germany, April 2016–March 2017 (n  = 314)

Resistance genes^a^	Total N = 314		Region/country of origin								P value^d^
			Pakistan/Afghanistan/Iran^b^ n = 183		Syria n = 68		Eritrea/Somalia^b^ n = 42		Other^c^ n = 21		
	n	%	n	%	n	%	n	%	n	%	
*bla*_TEM_^e^	125	40	69	38	34	50	14	33	8	38	NA
*bla*_OXA-1_^e^	43	14	22	12	8	12	10	24	3	14	NA
*bla*_CMY-4/42/58/59_ (AmpC)	4	1	4	2	0	0	0	0	0	0	NA
*bla*_SHV-1/11/75_	3	1	2	1	1	1	0	0	0	0	NA
**Total additional *bla* genes**	**175**	**56**	**97**	**53**	**43**	**63**	**24**	**57**	**11**	**52**	**0.53**
*aac(6')Ib-cr*	40	13	19	10	8	12	10	24	3	14	NA
*qnrA1*	1	0	0	0	1	1	0	0	0	0	NA
*qnrB1*	3	1	2	1	1	1	0	0	0	0	NA
*qnrB19*	3	1	1	1	2	3	0	0	0	0	NA
*qnrS1*	81	26	61	33	10	15	7	17	3	14	NA
**Total quinolone resistance genes**	**128**	**41**	**83**	**45**	**22**	**32**	**17**	**40**	**6**	**29**	**0.18**

ESBL: extended-spectrum β-lactamase; NA: not applicable.

^a ^Plasmid-mediated colistin resistance genes *mcr-1* or *mcr-2* were not found in any of the ESBL-producing Enterobacteriaceae.

^b^ Grouped according to United Nations Regions M49 standard [31].

^c^ Includes isolates from countries with less than five positive stool samples.

^d^ P value for chi-squared test testing the null hypothesis: ‘Presence of any β-lactamase/any plasmid-encoded quinolone resistance genes among all ESBL isolates is equally distributed over countries of origin’.

^e^ *bla*_TEM_ and *bla*_OXA-1-group_ genes were not completely sequenced.

Data are number (column %) of detected β-lactamase/plasmid-mediated quinolone resistance (PMQR) genes among ESBL-producing Enterobacteriaceae (total N), by country of origin, unless indicated otherwise. All *bla*_TEM_, *bla*_OXA-1-group_, *bla*_SHV_, *bla*_CMY_ and PMQR genes occurred in combination with ESBL genes (*bla*_CTX-M_).

### Characterisation of *E. coli* and its epidemic lineages

PCR screening revealed that *E. coli*-ST131 accounted for 24.4% of all ESBL-*E. coli*. Colonisation with epidemic ESBL-producing *E. coli*-ST131 clonal sublineages O16 and O25b varied by region of origin with O16 being predominantly from Syria and O25b from Eritrea and Somalia (Table 3). Phenotypic non-susceptibility to ciprofloxacin was detected in nearly all ST131-O25b (41/49) and many non-ST131 *E. coli* (67/234), but was rare in ST131-O16 (3/26). Supplementary Table S1 displays β-lactamase- and PMQR genes in *E. coli* and its epidemic lineage ST131.

**Table 3:** Proportion of *Escherichia coli*-ST131 in ESBL-producing *E. coli*, detected in 1,544 newly arrived asylum seekers arriving in Germany, by country/region of origin, April 2016–March 2017 (n = 309)

Characteristic	Total N = 309^a^		County/region of origin								P value
			Afghanistan/Pakistan/Iran^b^ n = 180		Syria n = 67		Eritrea/Somalia^b^ n = 41		Other^c^ n = 21		
	n	%	n	%	n	%	n	%	n	%	
Total *E. coli* ST131	75	24	34	19	23	34	14	34	4	19	0.029^d^
ST131-O16	26	8	11	6	13	19	2	5	0	0	0.022^e^
ST131-O25b	49	16	23	13	10	15	12	29	4	19	

ESBL: extended-spectrum β-lactamase; H0: null hypothesis.

^a^ The sequence type of one out of n = 310 *E. coli* isolated from asylums seekers could not be determined by PCR (ambiguous results).

^b^ Grouped according to United Nations Regions M49 standard [31].

^c^ Includes isolates from countries with less than five positive stool samples.

^d^ P value from chi-squared test testing H0: ‘The proportion of *E. coli* ST131 among all ESBL-producing *E. coli* (100%) is equally distributed over countries of origin’.

^e^ P value from chi-squared test testing H0: ‘Among ESBL-producing E. coli ST131 (100%), the proportions of *E. coli* ST131 serotypes O16 and O25b are equally distributed over countries of origin’.

Data are number (column %) of clonal lineages ST131-O16 and ST131-O25b among ESBL-producing *E. coli* (total N), by country of origin.

## Discussion

We conducted a large cross-sectional study on the prevalence of C-PE in newly arrived asylum seekers in Rhineland-Palatinate. Despite ample sample size and statistical power, our study did not find evidence supporting a higher C-PE prevalence in asylum seekers compared with that reported in the German community. Hence, it is unlikely that being a newly arrived asylum seeker from a country/region with increased prevalence of ESBL-PE colonisation alone, i.e. in the absence of morbidity that increases the likelihood of carbapenem use and/or hospitalisation before arrival in Europe, is a risk factor for C-PE colonisation and thus, should not be a reason for screening on admission to regular hospital wards.

A recent study reported C-PE colonisation in six of 290 asylum seekers (2.1%) who were screened upon hospital admission in Germany [16], which is contrary to what we found. The difference, however, could be explained by the different populations studied, with results from the hospital-based study likely influenced by patients with pre-morbidities and associated risk of C-PE colonisation, and therefore not representative for the overall population. Such differences re-inforce the need for both, community-based prevalence studies and an individual risk assessment upon hospital admission.

We found that the prevalence of ESBL-PE (i.e. non-C-PE) colonisation in newly arrived asylum seekers exceeds that of the population residing in Germany, which is assumed to be 2–6% in the community [17,32] and slightly higher (7–10%) in hospitalised patients [33,34]. With more than 29% of asylum seekers from Afghanistan, Pakistan and Iran, 20% from Syria and 12% from Eritrea and Somalia testing positive for ESBL-PE colonisation, the prevalences detected in our study are in line with estimates for south-east Asia (22%, 95% CI 7-44), eastern Mediterranean (15%, 95% CI 4-31), and Africa (22%, 95% CI 5-47) published by Karanika et al. [35] who combined the evidence of research on ESBL prevalence in 28,909 community dwellers in their large meta-analysis.

On the other hand, our estimates are considerably lower than those reported in a systematic review of ESBL colonisation prevalence in long-distance travellers returning to Europe; prevalence’s were reported to be over 70% in travellers returning from south- and south-east Asia [4]. Travellers’ diarrhoea and antibiotic use, both very common in travellers, were identified as risk factors for ESBL-PE colonisation [36]. The majority of travellers returning from long-distance travel clear ESBL-PE colonisation within the first month, with less than 10% still being colonised after 3 months [37]. Hence, apart from geographic variation in the colonisation risk, a predominance of ground- vs air-travel could explain the lower ESBL-PE colonisation prevalence observed in our sample of asylum seekers compared with long-distance travellers.

From a public health point of view, this puts our findings into perspective. First, considering the large number of air travellers returning to Europe from high prevalence countries each year, ESBL-PE faecal colonisation in asylum seekers is probably a minor contributor to the overall burden of ESBL-PE import. Second, as observed in travellers [37], ESBL-PE colonisation in asylum seekers is likely temporary. Thus the prevalence of colonisation should decrease to that of the population living in the host country after a few months. To confirm the latter hypothesis, research on the duration and risk factors of faecal ESBL-PE carriage in newly arrived asylums seekers is needed.

A recent review discussing enhanced contact precautions for all in-patients colonised with ESBL-producing *E. coli* concluded that the evidence base for their implementation is ‘rather weak’ [38] and currently these are not recommended by European guidelines [39]. In Germany, screening for ESBL-PE other than CPE is currently not recommended upon hospital admission and enhanced infection control measures for patients colonised with these bacteria are only recommended on high-risk wards (i.e. neonatology, haematology and intensive care units) [40]. Hence, our findings on ESBL-PE (i.e. non-C-PE) colonisation in newly arrived asylum seekers being predominantly caused by *E. coli*, do not warrant additional infection control measures. However, they may help to guide the choice of diagnostic tests, calculated antibiotic therapy, as well as the planning of invasive procedures in newly arrived asylum seekers.

We observed that sex modified the effect of age on ESBL-PE colonisation prevalence. The odds of ESBL-PE colonisation were highest in young males under 10 years and between the age of 30 and 39 years and in females between 20 and 39 years. The latter finding has also been described in other studies [41] and could potentially be explained by a higher incidence of urinary tract infections and associated antibiotic therapy in women of reproductive age.

Molecular analyses of ESBL-PE isolated in this study showed a dominant proportion of CTX-M-15 followed by CTX-M-27. CTX-M-15 is the most prevalent ESBL determinant worldwide and was also reported in up to 50% of ESBL-PE positive stool samples from Germany [17,42,43]. We found 24% of epidemic lineage *E. coli*-ST131 among ESBL-PE of asylum seekers, which is comparable to proportions in isolates from community dwellers, ambulatory and hospitalised patients in Germany [43]. From a public health point of view, this is relevant, since *E. coli*-ST131 and particularly the sublineage ST131-O25b is more virulent [44] and known to cause large outbreaks in both, the community and healthcare setting [45,46].We further observed that *E. coli*-ST131 mainly produced CTX-M-15 and CTX-M-27 (Supplementary Table S1). Of note, CTX-M-27 accounted for a third of all ESBL genes harboured by ST131 and half of those in ST131-O25b. The finding of CTX-M-27 in ESBL-PE from asylum seekers from all geographic regions supports the notion of its successful worldwide expansion, as proposed by Matsumura et al. [47]. Our findings support other studies that propose human travel as a main driver for the temporal and geographical shift in CTX-M-producing Enterobacteriaceae [48] and the *E. coli*-ST131 epidemic lineages [49]; a public health response that goes beyond targeting asylum seekers is now awaited.

This study has some limitations. First, precise information on travel routes and time would have been desirable, but could not be collected as stool samples from routine screening were used. However, this does not impact the main finding of a low prevalence of C-PE colonisation among asylum seekers in Germany. Second, we have no information regarding recent contact to the healthcare setting (e.g. hospital) or use of antibiotics. With regards to C-PE prevalence, our main outcome of interest, inclusion of ‘pre-morbid asylum seekers’ with an increased risk of antibiotic intake and/or hospitalisation compared to ‘community dwellers’ would lead to an overestimation. Therefore, we assume that this information bias has not affected our 0% prevalence finding. Finally, like other studies on the import of ESBL-PE and C-PE to Europe through international travel [3], we were not set up to detect OXA-48-like producers without ESBL phenotype through the use of ESBL screening agar.

In summary, our study detected no faecal colonisation with C-PE in 1,544 newly arrived asylum seekers. These data support the notion that being an asylum seeker from a country/region with increased ESBL-PE colonisation prevalence alone is not an indicator for C-PE colonisation upon arrival in Germany and thus not a reason for pre-emptive screening or isolation upon hospital admission.

## Note

*This designation is without prejudice to positions on status, and is in line with UNSCR 1244/1999 and the ICJ Opinion on the Kosovo declaration of independence.

## Supplementary Data

130000 Supplementary Table S1
