# Supplementary material for: No evidence of carbapenemase-producing Enterobacteriaceae in stool samples of 1,544 asylum seekers arriving in Rhineland-Palatinate, Germany, April 2016 to March, 2017
Source: Euro Surveill. 2019 Feb 21;24(8):1800030. doi: 10.2807/1560-7917.ES.2019.24.8.1800030 (PMC6446954; doi:10.2807/1560-7917.ES.2019.24.8.1800030)
Supplement: Supplementary Table S1 [file 1800030_ZANGER_SupplementaryTable.pdf]

"This supplementary material is hosted by Eurosurveillance as supporting information alongside the article [No evidence of carbapenemase-producing Enterobacteriaceae in stool samples of 1,544 asylum seekers arriving in Rhineland-Palatinate, Germany, April 2016 to – March 2017] on behalf of the authors who remain responsible for the accuracy and appropriateness of the content. The same standards for ethics, copyright, attributions and permissions as for the article apply. Eurosurveillance is not responsible for the maintenance of any links or email addresses provided therein."

Supplementary Table 1: β-lactamase- and plasmid-mediated quinolone resistance genes in epidemic lineages of *Escherichia coli*, detected in 1,544 asylum seekers arriving in Germany, 2016-2017

**Resistance gene**	***ESBL-producing E. coli***			
	**Non-ST131 n=234**	**ST131**		**Total N=309[a]**
		**ST131-O16 n=26**	**ST131-O25b n=49**	
CTX-M-15 ESBL	199	21	24	244
CTX-M-27 ESBL	6	0	24	30
CTX-M-1 ESBL	5	2	0	7
CTX-M-3 ESBL	7	1	0	8
CTX-M-55 ESBL	5	1	0	6
SHV-12 ESBL	4	0	0	4
CTX-M-14 ESBL	3	1	0	4
CTX-M-24 ESBL	2	0	0	2
CTX-M-9 ESBL	1	0	0	1
CTX-M-36 ESBL	0	0	1	1
CTX-M-32 ESBL	1	0	0	1
CTX-M-17 ESBL	1	0	0	1
Total ESBL genes	234	26	49	309
TEM-like[b]	100	17	6	123
OXA-1-group[b]	27	0	16	43
CMY-4/-42/-58/-59	3	0	1	4
Total additional β-lactamase genes	130	17	23	170
aac(6')Ib-cr	24	0	16	40
qnrA1	1	0	0	1
qnrB1	0	0	0	0
qnrB19	3	0	0	3
qnrS1	77	3	1	81
Total quinolone resistance genes	105	3	17	125

ESBL: extended-spectrum β-lactamase

Data are number of β-lactamase / plasmid-mediated quinolone resistance (PMQR) genes in E. coli and its ST131 lineages. All *bla*$_{TEM}$, *bla*$_{OXA-1}$-group, *bla*$_{SHV}$, *bla*$_{CMY}$ and PMQR genes occurred always in combination with ESBL genes (*bla*$_{CTX-M}$).

[a] The sequence type of one out of n = 310 *E. coli* isolated from asylums seekers could not be determined by PCR (ambiguous results).

[b] *bla*$_{TEM}$ and *bla*$_{OXA-1}$-group genes were not completely sequenced
